# Influence of duration of preoperative treatment with phenoxybenzamine and secretory phenotypes on perioperative hemodynamics and postoperative outcomes in pheochromocytoma and paraganglioma

**DOI:** 10.3389/fendo.2023.1139015

**Published:** 2023-04-19

**Authors:** Yao Yao, Ying Guo, Jing Fan, Tianxin Lin, Lin Wang, Shaoling Zhang

**Affiliations:** ^1^ Department of Endocrinology, Sun Yat-sen Memorial Hospital, Sun Yat-sen University, Guangzhou, China; ^2^ Department of Urology, Sun Yat-sen Memorial Hospital, Sun Yat-sen University, Guangzhou, China; ^3^ Department of Pathology, Sun Yat-sen Memorial Hospital, Sun Yat-sen University, Guangzhou, China

**Keywords:** pheochromocytoma, paraganglioma, phenoxybenzamine, preparation duration, secretory phenotype, intraoperative hemodynamic instability, postoperative outcomes

## Abstract

**Objectives:**

Resection of pheochromocytoma and paraganglioma (PPGL) carries risks with perioperative hemodynamic instability. Phenoxybenzamine (PXB) is a commonly used α-blockade to prevent it. It is unclear whether lengthening the preoperative duration of PXB is better for hemodynamic stability and postoperative outcomes. Furthermore, different types of catecholamines have varying effects on perioperative hemodynamics. Thus, our study aimed to investigate the impact of the duration of preoperative preparation with PXB and secretory phenotypes of the patients on intraoperative hemodynamic stability and postoperative complications in PPGL.

**Methods:**

Between Dec 2014 and Jan 2022, 166 patients with PPGL were operated on by the same team at Sun Yat-sen Memorial Hospital. They were divided into group A(1-14d), Group B(15-21d), and Group C(>21d) based on the duration of management with PXB and into the adrenergic and the noradrenergic phenotype group based on secretory profiles. Data on intraoperative hemodynamics and postoperative outcomes were collected and compared among groups.

**Results:**

A total of 96 patients occurred intraoperative hemodynamic instability, and 24 patients had 29 postoperative complications related to the surgery. Among the 145 patients treated with PXB, no significant differences were found in the cumulative time outside the target blood pressure(6.67%[0-17.16%] *vs.* 5.97%[0-23.08%] *vs.* 1.22%[0-17.27%], *p*=0.736) or in the median total HI-score(42.00[30.00-91.00] *vs.* 89.00[30.00-113.00] *vs.* 49.00[30.00-93.00], *p*=0.150) among group A(n=45), B(n=51) and C(n=49). Multivariate analysis demonstrated that the level of plasma-free metanephrine(MN) was an independent risk factor for intraoperative hemodynamic instability. And the median cumulative time outside of the target blood pressure in the adrenergic phenotype group was significantly greater than that in the noradrenergic phenotype group(8.17%[0-26.22%] *vs.* 1.86%[0-11.74%], *p*=0.029). However, the median total HI-score(99.50[85.00-113.25] *vs.* 90.00[78.00-105.00], *p*=0.570) and postoperative outcomes showed no differences between the two groups.

**Conclusions:**

A preoperative duration of nearly 14 days with PXB is sufficient for ensuring intraoperative hemodynamic stability in PPGL. And lengthening the preparation duration may not provide additional benefits in the era of widespread application and advanced techniques of laparoscopic surgery. Additionally, patients with the adrenergic phenotype are more prone to intraoperative hemodynamic instability than the noradrenergic phenotype. Thus, more attention should be given to the adrenergic phenotype during surgery.

## 1 Introduction

Pheochromocytoma and paraganglioma (PCC and PGL, collectively referred to as PPGL) are rare neuro-endocrine tumors with an estimated incidence of 0.6 to 8.3 cases per 100,000 person-years depending on the population studied (1). While they are rare, they can be lethal if not properly diagnosed and managed. In some cases, these tumors can cause severe or life-threatening episodes of hypertension due to excessive catecholamines including epinephrine, norepinephrine, and dopamine, which can lead to complications such as stroke, heart attack, or organ damage (2).

Generally, surgical removal of the tumor is the only effective treatment for PPGL (3). But resection of PPGL is associated with a high risk for perioperative hemodynamic instability, and even crises such as hypertensive emergency, malignant arrhythmia, and multiple organ dysfunction. Over the past few decades, several studies have shown that the use of α-blockade, particularly phenoxybenzamine, can significantly reduce the probability of perioperative mortality in patients undergoing resection of PPGL (4–8). These studies have demonstrated that the use of phenoxybenzamine has reduced the probability of perioperative mortality from 40% to less than 3% (7, 8). In addition, the Endocrine Society Clinical Guidelines in 2014 recommended the use of a-adrenergic receptor blockers, at least 7 days preoperatively, to reduce the risk of unpredictable instability in blood pressure during surgery (9).

Until now, there is no authoritative standardized duration of preoperative preparation. The 2021 National Comprehensive Cancer Network (NCCN) Clinical Practice Guidelines recommend a period of 7-14 days, while the Chinese Expert Consensus in 2021 recommend no less than two weeks (1, 10, 11). The actual duration of preoperative preparation time in practice also varies widely, from 2-13 weeks (12–15). However, it is important to note that there is a lack of prospective research on the impact of extending the preparation time on hemodynamic instability during the perioperative period. While one retrospective study concluded that extending the preparation time does not appear to be beneficial (13).

Also, the specific pattern of tumor catecholamine secretion can have a significant impact on hemodynamics, ranging from biochemically and clinically silence to severe hemodynamic instability caused by excessive catecholamine secretion (12, 16). Eisenhofer and colleagues proposed that tumors with a noradrenergic phenotype may be derived from less mature chromaffin cells and have a continuous secretion mode, which can lead to a less responsive and sustained effect on hemodynamics. In contrast, tumors with an adrenergic phenotype may be more sensitive to various stimuli and have a more immediate and transient effect on hemodynamics (17). So, different phenotypes lead to different biological effects on hemodynamics.

Finally, there are no unified criteria for hemodynamic instability. In this study, multiple indices including hemodynamic instability score(HI-score, **Supplement Table 1**) were applied to evaluate the incidence rate and the severity of hemodynamic instability. The HI-score is a scoring tool calculated as a weighted continuous measure ranging from 0-160 scores to define the overall degree of hemodynamic instability. It covers three aspects of hemodynamic variables (including systolic blood pressure(SBP), mean arterial pressure(MAP), heart rate(HR)), intravenous volume therapy, and intravenous administration of vasoactive medication, which represent both the fluctuation of hemodynamics as well as clinical measures to restore stability. This scoring system may be useful in assessing the severity of hemodynamic instability and in guiding clinical decision-making for appropriate management.

Therefore, the aim of our study is to investigate the optimal duration of preoperative preparation and the influence of secretory phenotypes on surgery outcomes in patients with PPGL using multiple indices.

## 2 Patients and methods

### 2.1 Patients and characteristics

**Figure 1:**
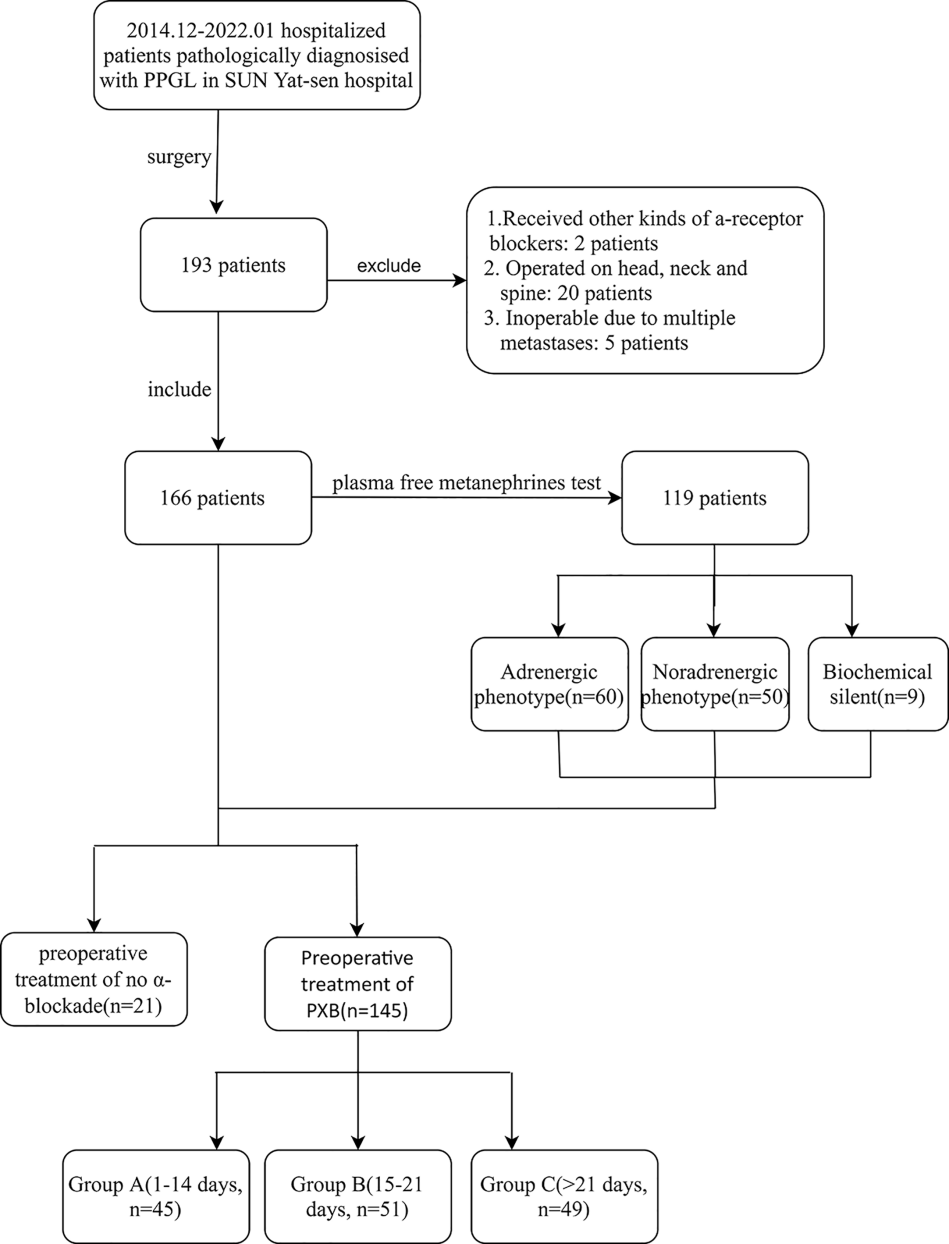
Inclusion and exclusion of subjects. PPGL, pheochromocytoma and paraganglioma; PXB, phenoxybenzamine; metanephrines, metanephrine and normetanephrine; Group A, B and C are divided according to the duration of preoperative management of PXB.

A total of 192 consecutive hospitalized patients who underwent surgery and were pathologically diagnosed with PPGL at Sun Yat-sen Hospital between December 2014 and January 2020 were included in the study. Two patients who received α-blockade other than PXB before surgery, twenty patients who had operations on the spine or neck other than abdomen, and five cases with a large amount of data missing were excluded. Finally, 166 patients were enrolled who underwent laparoscopic or laparotomy resection of their tumors (**Figure 1**). The majority of patients underwent at least one biochemistry test prior to surgery, including adrenaline, norepinephrine, metanephrine(MN), normetanephrine(NMN) of plasma or urinary, and total vanillylmandelic acid excretion in urine for 24 hours. Metanephrine and normetanephrine are collectively referred to as metanephrines (MNs). Of which, samples of plasma-free MNs were collected from patients in the supine position into anticoagulant tubes containing heparin, centrifuged on ice(4°C) as soon as possible, and then stored at −80°C until assayed which was measured with liquid chromatography-tandem mass spectrometry(LC-MS/MS) by KingMed Diagnostics in Guangzhou, China. All patients were required to avoid caffeinated beverages intake overnight and acetaminophen intake for seven days.

We collected the patients’ characteristics, tumor characteristics, hormonal workup, blood pressure, HR, and medication at diagnosis. The preoperative duration was defined as the continuous time that passed between the first dose of PXB and the date of the surgical procedure, and the cumulative dose of PXB was recorded. The study also recorded the American Society of Anesthesiologists’ physical status classification(ASA class, **Supplement Table 2**), the method and duration of surgery and anesthesia, as well as postoperative complications (18) which occurred during the hospitalization of the subjects.

The study defined the secretory phenotype of PPGL based on the patient’s hormonal workup, specifically the levels of plasma-free MNs. The noradrenergic phenotype with predominantly noradrenaline secretion manifested by a marked elevation of plasma-free NMN, along with normal plasma-free MN or by increases of less than 5% for MN to the combined increments of both metabolites. Conversely, the adrenergic phenotype with predominantly adrenaline secretion was defined as an increase of plasma-free MN above the upper reference limits and associated increments of more than 5% for MN to the combined increments of both metabolites (17, 19, 20). In cases where neither MN nor NMN was increased to the upper limit of the reference value by at least two tests, the study defined the patient as having a biochemical silent phenotype.

Hypertension was defined as increased SBP over 140 and/or diastolic blood pressure(DBP) over 90 mmHg or the use of antihypertensive medication, otherwise it was defined as normotension. Diabetes was defined according to the American Diabetes Association (ADA) guidelines updated in 2017 (21).

### 2.2 Preoperative management

In our center, normotensive PPGL with hormonal overproduction and hypertensive PPGL with hormonally inactive were both treated with α-blockade as a preparation for surgery. On the other hand, for normotensive PPGL with hormonally inactive secretion, we did not routinely prescribe α-blockade to these patients but informed surgeons and anesthesiologists to prepare hemodynamic management of PPGL during operation. When patients with PPGL were clinically diagnosed, PXB as the first option of α-blockade was managed with patients, and the initial dose was 10 mg orally once daily, gradually increased according to blood pressure until controlling to less than 130/80 mmHg or when the patient cannot tolerate the side effects of PXB(such as tachycardia, postural hypotension, and nasal mucosa). In our center, achieving the target blood pressure without any discomfort is a necessity to determine the length of treatment. The target values of sufficient preparation for surgery were defined as follows: (1)target blood pressure(BP): seated BP <130/80 mmHg, standing SBP >90 mmHg; target HR: seated HR of 60~70 beats per minute(bpm), standing HR of 70~80bpm (9); no obvious orthostatic hypotension; (2)blood volume recovery: reduced hematocrit, weight gain, the extremity warmth, reduced sweating, nasal congestion, and improved microcirculation. Other hypertensive drugs such as β-blockades and calcium channel blockers may be added on if BP and HR do not reach the targets. The treatment approach was modified according to each patient’s age and physical condition.

According to the duration of PXB, 145 patients were divided into three groups: Group A (1-14 d), Group B (14-21 d), and Group C(>21d). All patients were recommended on a high-sodium diet and two liters of 0.9% saline was administered intravenously daily during the last 48 hours before surgery.

### 2.3 Intraoperative details

The surgery was all conducted by the same team of highly skilled and experienced anesthesiologists and surgeons. The type of surgery recommended for each patient depended on the size and invasiveness of the tumor. Laparoscopic surgery was recommended for small and non-invasive tumors, while open surgery was recommended for larger or more invasive tumors. Two laparoscopic approaches were used: the lateral transabdominal approach and the posterior retroperitoneal approach.

In our center, air inhalation combined with intravenous general anesthesia was the preferred anesthesia approach supplemented by epidural anesthesia and controlled hypotension. During surgery, all patients received an intra-arterial catheter for continuous BP and HR monitoring which were automatically recorded every five minutes by computer. The use of sodium nitroprusside, phentolamine, nitroglycerin, and esmolol for managing hypertension and arrhythmia is consistent with current practice guidelines (22). If hypotension occurs following surgical removal of the tumor, fluid infusions and vasoactive drugs such as norepinephrine, epinephrine, and dopamine can be administered.

Intraoperative hemodynamic instability was defined as at least one of the four marks: (1) SBP >160 mmHg; (2)MAP <60 mmHg; (3)HR >100 bpm; (4)HR <50 bpm. The primary outcome was the cumulative time between the incision and suturing of the incision expressed as a percentage. The HI-score as a secondary outcome is higher in patients with more unstable hemodynamics. The three components and attributed points are shown in **Supplement Table 1**. For hemodynamic stability, SBP_max_, MAP_min_, HR_max_, HR_min_, fluid intake, and the use of hypertensive and vasoactive drugs were also recorded. In addition to postoperative complications that occurred during hospitalization, the length of hospital and intensive care unit stays were included in postoperative outcomes. Postoperative hypotension was defined as a decrease in SBP below 90 or the use of vasoactive drugs to maintain it.

## 3 Statistical analysis

All statistical tests were performed with SPSS 25.0 (IBM Corporation, Armonk, NY, USA), and statistical significance was set at *p*<0.05. The mean and standard deviation were recorded for normally distributed continuous variables and the median and range (or IQR or sometimes 95% CI) for non-normally distributed variables. A t-test was used to compare normally distributed samples when the homogeneity of variance was met, and a corrected t-test was used when the homogeneity of variance was not met. The Mann-Whitney U test was used for quantitative data with non-normal distribution and the Kruska - Wallis test for variables with non-normal distribution. The chi-square test was used to test the distribution of categorical variables and the F-test was used for continuous variables. A univariate and multivariate linear regression analysis is performed in order to confirm the presence of independent predictors of intraoperative hemodynamic instability.

## 4 Result

### 4.1 General characteristics

In the study, 166 patients(77 men and 89 women, average age at diagnosis: 48.1 years) underwent resection of PPGL at Sun Yat-sen Memorial Hospital of Sun Yat-sen University (**Table 1**). As reported in the pathology report, the median diameter of the tumors was 4.8 cm (3.8-6.5 cm). A total of 127 tumors were located in the adrenal medulla, whereas 39 tumors originated from sympathetic paraganglia in extra-adrenal areas. A large majority of patients(n=154) underwent laparoscopy by either lateral transperitoneal(n=13) or posterior retroperitoneal(n=141). There were only 12 patients who underwent a laparotomy due to unknown origins of tumors prior to surgery or difficulty in dissecting tumors under laparoscopy. Four patients were converted from laparoscopy to laparotomy due to difficulties with dissection. The median duration of operation and anesthesia is 125(81.5-190) minutes and 280(225-333.5) minutes, respectively.

**Table 1:** General characteristics of 166 patients operated on for pheochromocytoma or paraganglioma.

Characteristics	All patients(n=166)
Sex ratio(male/female)	77/89
Median age at diagnosis(years)	48.1 ± 15.4
Mean BMI(kg/m^2^)	22.24 ± 3.30
Patients with diabetes	48/118
Patients with paroxysmal symptoms	82/84
Median tumour diameter(cm)	4.80(3.80-6.50)
Tumor location(adrenal/extra-adrenal)	127/39
SBP at diagnosis(mmHg)	130.50(119.75-148.25)
DBP at diagnosis(mmHg)	82.00(73.00-93.00)
HR at diagnosis(bpm)	81.00(75.00-93.00)
Normotension/hypertension at diagnosis	44/122
Cases of treating with PXB(preoperative)	145/166
Cases of treating with β -blockades(preoperative)	52/166
Phenotype(adrenergic/noradrenergic/Biochemically silent)	60/50/9
Plasma free MN(nmol/L)	0.89(0.20-3.95)
Plasma free NMN(nmol/L)	7.51(2.03-14.78)
^a^Recurrence	15
^b^Malignancy	11
ASA class II/III/IV(n)	22/96/13
^c^Anesthesia duration(minutes)	280.00(225.00-333.50)
Open surgery/laparoscopy(n)	12/154
Transabdominal/retroperitoneal(n)	25/141
^d^Surgery duration(minutes)	125.00(81.50-190.00)

Data are shown as mean ± SD, medians, and [P5-P95] intervals or proportions. BMI, body mass index; SBP, systolic blood pressure; DBP, diastolic blood pressure; HR, heart rate; PXB, phenoxybenzamine; CCBs, calcium channel blockers; MN, metanephrine; NMN, normetanephrine; ASA, American Society of Anesthesiologists.

a Recurrence was defined as local relapse, new chromaffin tumor, or metachronous metastases found by image.

b Magnliance is defined as metastasis occurring where chromaffin tissue is normally not found.

c Time from induction of anesthesia until suturing of the incision.

d Time from incision until suturing of the incision.

There were 145 patients who were managed with PXB before surgery. The participants were divided into three groups based on the length of time they received PXB preoperatively: group A (1-14 days, n=45), group B (14-21 days, n=51), and group C (>21 days, n=49) (**Table 2**). Neither demographic characteristics such as gender, tumor size nor plasma-free MNs were significantly different among the three subgroups (**Table 2**). One group of patients(n=21, 16 PGLs and 5 PCCs) received no PXB before surgery because there was no suspicion of PPGL involvement. Among them, 11 patients with retroperitoneal masses and two others located either in the duodenum or the pancreas were not tested for plasma-free metanephrines during their initial surgical evaluation, which resulted in them not receiving PXB. The remaining eight patients had combined adrenal masses that were not considered the diagnosis of PPGL and were not treated with PXB for various reasons.

**Table 2:** Characteristics of three groups with PPGL according to the duration of administration of PXB.

	Group A (1-14days, n=45)	Group B (15-21days, n=51)	Group C (>21days, n=49)	H/F/*χ* ^2^	*p* value
Sex ratio(male/female)	21/24	22/29	24/25	0.346	0.841
BMI(kg/m2)	22.76 ± 3.70	21.87 ± 2.91	22.29 ± 2.96	0.637	0.531
Mean age at diagnosis(years)	45.2 ± 15.4	52.7 ± 14.2^£^	46.3 ± 15.9	3.444	0.035^*^
Mean tumor diameter (cm)	4.70(3.35-6.00)	4.80(3.90-7.13)	4.60(4.00-6.35)	1.538	0.463
Plasma free MN(nmol/L)	0.85(0.19-2.30)	1.40(0.27-4.28)	0.90(0.20-5.72)	0.870	0.647
Plasma free NMN(nmol/L)	6.72(1.56-11.18)	7.70(2.34-17.55)	8.79(2.53-18.31)	3.083	0.214
Median time of administration of PXB(days)	13.00(10.00-13.50)	18.00(16.00-20.00)	30.00(25.50-35.00)	121.646	<0.001^*^
Total dosage of PXB(g)	0.37(0.24-0.52)	0.66(0.50-0.88)	1.04(0.62-1.55)	60.083	<0.001^*^
Surgical approach (laparoscopy/open surgery)	43/2	47/4	47/2	0.821	0.663
Transabdominal/retroperitoneal	40/5	30/9	46/3	0.789	0.674
^a^Surgery time(minutes)	95.00(72.50-167.50)	125.00(80.00-190.00)	145.00(100.00-207.50)	0.817	0.665
ASA class II/III/IV(n)	9/32/4	7/41/3	6/40/3	0.328	0.849
^b^Anesthesia duration(minutes)	270.00(210.00-320.00)	280.00(220.00-360.00)	291.00(212.00-330.00)	1.537	0.464
Preoperative SBP(mmHg)	134.00(116.50-149.50)	138.00(121.00-156.00)	131.00(120.00-140.00)	1.532	0.465
Preoperative DBP(mmHg)	83.00(76.50-94.00)	82.00(73.00-94.00)	85.00(75.00-93.00)	0.008	0.961
Preoperative HR(bpm)	80.00(73.00-89.50)	84.00(72.00-93.00)	82.00(77.00-95.00)	0.513	0.774

Data are shown as mean ± SD, medians, and [P5-P95] intervals or proportions. PPGL, pheochromocytoma and paraganglioma; PXB, phenoxybenzamine; BMI, body mass index; MN, metanephrine; NMN, normetanephrine; SBP, systolic blood pressure; DBP, diastolic blood pressure; HR, heart rate; bpm, beats per minutes; ICU: intensive care unit; ASA, American Society of Anesthesiologists. *Statistically significant; ^$^:indicates two groups with a value significantly different from each other with such a symbol. ^£^:indicates the subgroups with values significantly different from the other subgroups.

a Time from incision until suturing of the incision.

b Time from induction of anesthesia until suturing of the incision.

There were 119 patients whose plasma-free MN levels had been measured (**Table 3**). The majority of the participants, comprising 110 individuals, were found to secrete catecholamines. Out of these patients, 60 exhibited the adrenergic phenotype, while 50 presented with the noradrenergic phenotype. Interestingly, only a small minority of patients (9 individuals, including 5 PGLs and 4 PCCs) were considered biochemically silent. The adrenergic phenotype was characterized by a great number of patients (51 individuals) who had elevated levels of MN and NMN. In contrast, the noradrenergic phenotype was more frequently observed in patients who were younger at diagnosis, with an average age of 54.1 ± 13.3 compared to 41.3 ± 15.1 in the adrenergic phenotype (*p*<0.001). Tumors originating from extra-adrenal sites also tended to be associated with the noradrenergic phenotype (*p*<0.001). Additionally, the noradrenergic phenotype was correlated with a smaller tumor diameter than the adrenergic phenotype (*p*=0.036). In addition, a few patients did not have the results of plasma-free MNs. Some patients underwent alternative biochemical diagnostic tests, including plasma catecholamines and VMA excretion in urine over 24 hours. These tests are also indicative of PPGL. A number of patients were initially diagnosed with PPGL at other hospitals and were referred to our center for surgical treatment. Some patients were part of the 21 patients who did not receive PXB treatment, while the remaining patients did not for other reasons such as experiencing a pathological biopsy diagnosis and lost records.

**Table 3:** Characteristics of patients with PPGL in relation to their biochemical profile.

	The adrenergic phenotype(n=60)	The noradrenergic phenotype(n=50)	*t*/*Z*/*χ* ^2^	*p*-value
Sex ratio(male/female)	27/33	21/29	0.100	0.752
Age at diagnosis(years)	21.9 ± 2.3	22.5 ± 4.1	-0.768	0.446
BMI(kg/m2)	54.07 ± 13.25	41.26 ± 15.1	4.736	<0.001^*^
Tumor size(cm)	5.00(4.00-6.88)	4.50(3.50-6.00)	-2.096	0.036^*^
Tumor location(adrenal/extra-adrenal)	56/4	33/17	13.191	<0.001^*^
Plasma free MN(nmol/L)	4.02(2.14-11.82)	0.23(0.16-0.42)	-8.490	<0.001^*^
Plasma free NMN(nmol/L)	6.86(1.94-13.18)	10.35(6.57-17.60)	-2.989	<0.004^*^
Preoperative SBP(mmHg)	136.97 ± 24.00	138.76 ± 24.70	-0.385	0.701
Preoperative DBP(mmHg)	80.00(71.00-92.50)	86.50(79.00-97.25)	-1.974	0.052
Preoperative HR(bpm)	80.00(75.75-92.75)	84.00(77.50-96.50)	-0.861	0.389
Preoperative duration of PXB treatment(days)	18.00(13.00-26.25)	16.00(13.00-27.25)	-0.629	0.530
Total dose of PXB(g)	0.60(0.45-1.03)	0.64(0.46-0.90)	-0.006	0.952
Surgery approach (laparoscopy/open surgery)	58/2	49/1	0.187	0.665
Transabdominal/retroperitoneal	56/4	44/6	0.404	0.525
^a^Surgical time(minutes)	120.00(80.00-163.75)	104.50(80.00-172.50)	-0.673	0.501
ASA class II/III/IV(n)	12/44/4	7/39/4	0.617	0.432
^b^Anesthesia duration(minutes)	273.50(216.25-320.00)	270.00(213.00-330.00)	-0.339	0.734

Data are shown as mean ± SD, medians, and [P5-P95] intervals or proportions. PPGL, pheochromocytoma and paraganglioma; PXB, phenoxybenzamine; BMI, body mass index; MN, metanephrine; NMN, normetanephrine; SBP, systolic blood pressure; DBP, diastolic blood pressure; HR, heart rate; ICU: intensive care unit; ASA, American Society of Anesthesiologists; bpm, beats per minutes. *Statistically significant.

a Time from incision until suturing of the incision.

b Time from induction of anesthesia until suturing of the incision.

### 4.2 Perioperative hemodynamic outcomes

During our study, we noted hemodynamic instability during surgery in 85 patients, and 29 complications related to tumors occurred in 24 patients. Postoperative adverse events were primarily attributed to hypotension. A comprehensive list of all events is provided in **Table 4**.

**Table 4:** Postoperative complications.

Complications	Numbers
Hypotension and/or need vasoactive drugs for sustained blood pressure	11
Intestinal obstruction	1
Lung infection or wound infection	5
Vital organ failure (heart, lung, kidney, etc.)	5
Water-electrolyte disorders	1
Disseminated intravascular coagulation	1
Ischemic-hypoxic encephalopathy	1
Fracture	1
Atrial fibrillation	1
Adrenal crisis	2
Total number of cases	29

#### 4.2.1 Perioperative outcomes with PXB and no α-bloackade treatment

During the surgery, the median cumulative time outside the target blood pressure range was 5.56% in the PXB group, compared to 1.61% in the no-blockade group (*p*= 0.302). The median total HI-score was higher in the PXB group than in the no α-blockade group(46.00[30.00-105.00] *vs.* 30.00[6.00-39.00], *p*=0.004) (**Table 5**). Of which, the volume therapy and medication scores were higher in patients managed with PXB than those who were not. There were no significant differences in the cumulative time and frequency of SBP >160 mmHg, MAP <60 mmHg, HR >100 bpm, or HR <50 bpm between the two groups (**Table 5**). Additionally, postoperative outcomes did not differ between the two groups.

**Table 5:** The effect of α-blockade on perioperative hemodynamics of patients with PPGL.

	no α-blockade(n=21)	PXB(n=145)	*Z*/*χ* ^2^	*p* value
Intraoprative hemodynamics				
Outside the targets				
Frequency(n)	11/21	85/145	0.293	0.588
Cumulative time(%)	1.61(0-12.19)	5.56(0-17.52)	-1.033	0.302
SBP >160 mmHg				
Frequency(n)	5/21	45/145	0.455	0.500
Duration(%)	0(0-1.38)	0(0-3.77)	-0.584	0.560
MAP <60 mmHg				
Frequency(n)	5/21	28/145	0.036	0.849
Duration(%)	0(0-0.81)	0(0-0)	-0.275	0.783
HR >100 bpm				
Frequency(n)	6/21	59/145	1.172	0.279
Duration(%)	0(0-4.51)	0(0-8.93)	-1.071	0.284
HR <50 bpm				
Frequency(n)	1/21	6/145	0.017	0.896
Duration(%)	0(0-0)	0(0-0)	-0.119	0.906
SBP_max_(mmHg)	140.00(131.00-157.50)	145.00(130.00-165.00)	-0.297	0.768
MAP_min_(mmHg)	65.00(59.50-72.50)	68.00(60.00-73.00)	-0.339	0.734
HR_max_(bpm)	90.00(77.50-101.00)	92.00(80.00-110.00)	-0.978	0.328
HR_min_(bpm)	60.00(53.00-62.00)	61.00(55.00-70.00)	-1.366	0.172
Vasoactive drugs				
Frequency(n)	8/21	79/145	1.975	0.160
Number(n)	0(0-1.00)	1.00(0-1.00)	-1.634	0.102
Antihypertensive drugs				
Frequency(n)	15/21	89/145	0.792	0.374
Number(n)	1.00(0-2.00)	1.00(0-2.00)	-0.470	0.639
Blood loss(mL)	90.00(30.00-300.00)	175.00(30.00-387.50)	-0.977	0.329
Infusion volume(L)	1.75(1.50-2.50)	2.00(1.50-2.50)	-0.898	0.369
^a^Blood transfusion(mL)	0(0-0)	0(0-0)	-1.017	0.309
HI score	30.00(6.00-39.00)	46.00(30.00-105.00)	-2.844	0.004^*^
Hemodynamic variables	1.00(0-7.00)	4.00(0-10.00)	-0.853	0.394
Volume therapy	14.00(6.00-30.00)	30.00(14.00-30.00)	-2.536	0.011^*^
Medication	0(0-5.00)	5.00(0-75.00)	-1.976	0.048^*^
Postoperative outcomes				
Postoperative SBP(mmHg)	130.00(116.00-144.50)	124.00(110.00-140.00)	-0.613	0.540
Postoperative DBP(mmHg)	79.00(67.50-80.00)	72.00(68.00-80.00)	-0.302	0.762
Postoperative heart rate(bpm)	80.00(68.50-90.00)	80.00(70.00-90.00)	-0.129	0.897
Fluid intake during 24-48 hours after surgery(L)	2.60 ± 1.44	2.41 ± 0.95	-0.346	0.732
Cases of admitting to ICU	1/21	9/145	0.072	0.789
Length of the ICU stay(days)	0(0-0)	0(0-0)	-0.206	0.837
Postoperative hospitalization days	7.00(5.00-9.00)	7.00(5.00-8.00)	0.223	0.824
^b^Complications	3/21	21/145	0.001	0.981

Data are shown as mean ± SD, medians, and [P5-P95] intervals or proportions. PPGL, pheochromocytoma and paraganglioma; PXB, phenoxybenzamine; outside the targets means at least one of SBP >160 mmHg, MAP <60 mmHg, HR >100 bpm or/and HR <50bpm appears; SBP, systolic blood pressure; DBP, diastolic blood pressure; MAP, mean arterial pressure; HR, heart rate; bpm: beats per minute; ICU: intensive care unit; *Statistically significant; _max_: maximum; _min_: minimum.

a Transfusion of red blood cells and plasma.

b assessed according to the Common Terminology Criteria for Adverse Events.

#### 4.2.2 Perioperative outcomes according to the duration of PXB treatment

There were no significant differences among groups A, B, and C in the cumulative time outside the target blood pressure(6.67%[0-17.16%] *vs.* 5.97%[0-23.08%] *vs.* 1.22%[0-17.27%], *p*=0.736) or in the median total HI-score(42.00[30.00-91.00] *vs.* 89.00[30.00-113.00] *vs.* 49.00[30.00-93.00], *p*=0.150). The frequency and duration of MAP<60mmHg, as well as the proportion of patients admitted to the ICU were all lower in group C than in the other two groups (**Table 6**). No significant differences were found in the total length of hospital stay or complications between groups A, B, and C (**Table 6**). Neither peak SBP and HR, valley SBP and HR nor the use of antihypertensive and vasoactive drugs differed between the three groups.

**Table 6:** Perioperative hemodynamics of three groups with PPGL according to the duration of administration of PXB.

	Group A (1-14days, n=33)	Group B (15-21days, n=39)	Group C (>21days, n=37)	H/F/*χ* ^2^	*p-*value
Intraoprative hemodynamics					
Outside the targets					
Frequency(n)	28	32	25	1.765	0.414
Cumulative time(%)	6.67(0-17.16)	5.97(0-23.08)	1.22(0-17.79)	0.613	0.736
SBP >160 mmHg					
Frequency(n)	10	14	7	2.550	0.279
Duration(%)	0(0-4.20)	0(0-5.56)	0(0-0)	2.923	0.232
MAP <60 mmHg					
Frequency(n)	11	14	3^£^	8.400	0.015^*^
Duration(%)	0(0-0.76)	0(0-1.75)	0(0-0)^£^	8.196	0.017^*^
HR >100 bpm					
Frequency(n)	19	19	21	0.388	0.823
Duration(%)	0(0-9.09)	0(0-5.56)	0(0-13.25)	1.150	0.563
HR <50 bpm					
Frequency(n)	2	3	1	0.945	0.623
Duration(%)	0(0-0)	0(0-0)	0(0-0)	0.951	0.621
SBP_max_(mmHg)	145.56 ± 27.75	151.94 ± 23.09	146.20 ± 22.18	2.093	0.334
MAP_min_(mmHg)	67.00(59.50-73.50)	65.00(59.00-70.00)	69.00(62.00-73.15)	3.476	0.176
HR_max_(bpm)	90.00(80.00-110.00)	95.00(80.00-105.00)	94.00(82.00-110.00)	0.277	0.871
HR_min_(bpm)	60.00(50.00-70.00)	60.00(52.00-70.00)	65.00(58.50-78.00)	3.096	0.213
antihypertensive drugs					
Frequency(n)	22	33	24	3.316	0.191
Number(n)	1.00(0-2.00)	1.00(0-2.00)	1.00(0-2.00)	3.399	0.183
Vasoactive drugs					
Frequency(n)	23	35	31	3.205	0.201
Number(n)	1.00(0-1.00)	1.00(1.00-2.00)	1.00(0-1.00)	1.384	0.500
Blood loss(mL)	100.00(12.50-300.00)	175.00(20.00-362.50)	275.00(50.00-412.50)	4.538	0.103
Infusion volume(L)	2.00(1.50-2.50)	2.00(1.50-3.00)	2.00(1.50-2.50)	1.637	0.441
^a^Blood transfusion(mL)	0(0-0)	0(0-0)	0(-100.00)	1.226	0.542
HI-score	42.00(30.00-91.00)	89.00(30.00-113.00)	49.00(30.00-93.00)	3.792	0.150
Hemodynamic variables	4.00(0-12.00)	4.00(0-10.00)	4.00(0-6.00)	3.922	0.141
Volume therapy	30.00(22.50-30.00)	30.00(15.00-30.00)	14.00(14.00-30.00)^£^	15.013	0.001^*^
Medication	0(0-60.00)^$^	75.00(0-75.00)^$^	35.00(0-75.00)	6.389	0.041^*^
Postoperative outcomes					
Postoperative SBP(mmHg)	122.20 ± 20.55	127.41 ± 19.68	129.63 ± 22.62	2.955	0.228
Postoperative DBP(mmHg)	75.00(69.00-84.50)	70.00(65.00-80.00)	72.00(65.00-81.50)	1.414	0.493
Postoperative HR(bpm)	80.00(71.50-90.00)	78.00(68.00-87.00)	80.00(70.00-90.00)	1.315	0.518
Fluid intake during 24-48 hours after surgery(L)	2.30(1.68-3.00)	2.50(1.95-2.94)	2.41(1.63-2.95)	0.845	0.655
Cases of admitting to ICU	4	5	0^£^	3.279	0.070
Length of the ICU stay(days)	0(0-0)^$^	0(0-0)	0(0-0)^$^	4.876	0.087
Postoperative hospitalization days	7.00(5.00-8.00)	7.00(6.00-9.00)	6.00(5.00-8.00)	2.522	0.283
^b^Complications	4^£^	10	7	0.220	0.330

Data are shown as mean ± SD, medians, and [P5-P95] intervals or proportions. PPGL, pheochromocytoma and paraganglioma; PXB, phenoxybenzamine; outside the targets means at least one of SBP >160 mmHg, MAP <60 mmHg, HR >100 bpm and HR <50 bpm appears; SBP, systolic blood pressure; DBP, diastolic blood pressure; HR, heart rate; bpm, beats per minutes; HI-score, hemodynamic instability; ICU: intensive care unit; ASA, American Society of Anesthesiologists. *Statistically significant; ^$^:indicates two groups with a value significantly different from each other with such a symbol. ^£^:indicates the subgroups with values significantly different from the other subgroups.

a Transfusion of red blood cells and plasma.

b assessed according to the Common Terminology Criteria for Adverse Events.

#### 4.2.3 Characteristics of patients with hemodynamic instability

An analysis of 85 patients with intraoperative hemodynamic instability and 60 patients without showed that the former group had a larger mean tumor size(5.00[3.80-7.00] vs. 4.50[3.53-5.00], p=0.016), and had nearly five-fold higher excretion of MN than the latter group (**Supplement Table 4**). As expected, these patients had sustained longer mean anesthesia and hospital stays postoperatively (7.00[6.00-9.00] vs. 6.00[5.00-7.00], p<0.001), as well as greater use of antihypertensive and vasoconstrictive drugs compared to those who did not experience hemodynamic instability.

For 24 patients with postoperative complications (**Supplement Table 5**), neither secretory phenotypes nor duration of preoperative administration of PXB was significantly different from those without postoperative complications.

Multivariate analysis revealed that plasma-free MN was an independent risk factor for intraoperative hemodynamic instability during the resection of PPGL, while the tumor size or the duration of preoperative PXB treatment were not predictive factors for hemodynamic instability (**Table 7**).

**Table 7:** Predictive factors of perioperative hemodynamics instability for PPGL according to univariate and multivariate analysis.

Variables	Univariate analysis			^#^Multivariate analysis		
	Odds ratio 95%CI		*p*-value	Odds ratio 95%CI		*p*-value
Sex(male/female)	2.319	[1.180-4.555]	0.015^*^	1.575	[0.690-3.596]	0.281
Age at diagnosis(years)	2.319	[1.180-4.555]	0.502			
BMI(kg/m2)	1.007	[0.986-1.029]	0.154			
Paroxysmal symptoms	2.278	[1.160-4.474]	0.017*	1.827	[0.759-4.400]	0.179
Tumor size(cm)	0.908	[0.795-1.037]	0.016^*^	1.093	[0.871-1.371]	0.442
Tumor location(adrenal/extra-adrenal)	2.278	[1.160-4.474]	0.499			
Plasma MN(nmol/L)	1.237	[1.041-1.471]	0.002^*^	1.219	[1.042-1.426]	0.013^*^
Plasma NMN(nmol/L)	1.343	[0.571-3.160]	0.027^*^	1.025	[0.973-1.080]	0.345
Preoperative SBP(mmHg)	1.060	[1.007-1.115]	0.366			
Preoperative DBP(mmHg)	1.006	[0.992-1.021]	0.746			
Preoperative HR(bpm)	1.003	[0.983-1.024]	0.717			
Preoperative duration of PXB treatment(days)	0.996	[0.975-1.018]	0.421			
Total dose of PXB(g)	1.007	[0.990-1.024]	0.515			

PPGL, pheochromocytoma and paraganglioma; CI, confidence interval; BMI, body mass index; MN, metanephrine; NMN, normetanephrine; SBP, systolic blood pressure; DBP, diastolic blood pressure; HR, heart rate; bpm, beats per minutes; PXB, phenoxybenzamine; *statistically significant; ^#^variables which were significant at p<0.10 in univariate analysis were included into the multivariate analysis.

#### 4.2.4 Perioperative outcomes according to the secretory phenotypes

Due to the remarkable effects of plasma-free MNs on hemodynamic instability, we categorized the patients into the adrenergic phenotype group and the noradrenergic phenotype group. The adrenergic phenotype group had a significantly longer median cumulative time outside the target blood pressure compared to the noradrenergic phenotype group(8.17%[0-26.22%] *vs.* 1.86%[0-11.74%], *p*=0.029) (**Table 8**). Patients of the noradrenergic phenotype group had a higher heart rate(*p*=0.019) and longer duration of HR >100bpm(*p*=0.047) than those in the adrenergic phenotype group. However, the median total HI-score showed no significant difference between the adrenergic and noradrenergic phenotype group(46.50[30.00-110.50] *vs.* 56.00[30.00-105.00], *p*=0.570). There were no significant differences in the length of the postoperative hospital stay or ICU stays between the two groups, either.

**Table 8:** Perioperative hemodynamics of patients with PPGL in relation to their biochemical profile of PPGL.

	The adrenergic phenotype(n=51)	The noradrenergic phenotype(n=30)	*t*/*Z*/*χ* ^2^	*p*-value
Intraoprative hemodynamics				
Outside the targets				
Frequency(n)	42/60	26/50	3.744	0.053
Cumulative time(%)	8.17(0-26.22)	1.86(0-11.74)	-2.184	0.029^*^
SBP>160mmHg				
Frequency(n)	23/60	13/50	1.884	0.170
Duration(%)	0(0-6.25)	0(0-1.75)	-1.681	0.093
MAP<60mmHg				
Frequency(n)	13/60	10/50	0.046	0.831
Duration(%)	0(0-0)	0(0-0)	-0.135	0.893
HR>100 bpm				
Frequency(n)	30/60	17/50	2.853	0.091
Duration(%)	0(0-14.29)	0(0-2.47)	-1.990	0.047^*^
HR<50 bpm				
Frequency(n)	3/60	2/50	0.063	0.802
Duration(%)	0(0-0)	0(0-0)	-0.200	0.842
SBP_max_(mmHg)	153.13 ± 23.91	144.44 ± 24.99	-1.737	0.082
MAP_min_(mmHg)	67.50(60.00-70.00)	66.00(60.00-74.25)	-0.567	0.571
HR_max_(bpm)	99.50(85.00-113.25)	90.00(78.00-105.00)	-2.355	0.019^*^
HR_min_(bpm)	60.00(52.00-70.00)	60.50(54.75-70.00)	-0.187	0.852
Antihypertensive drugs				
Number(n)	41/60	28/50	1.774	0.183
Frequency(n)	1.00(0-2.00)	1.00(0-2.00)	-1.892	0.058
Vasoactive drugs				
Number(n)	34/60	24/50	0.822	0.365
Frequency(n)	1.00(0-1.00)	0(0-1.00)	-1.204	0.229
Blood loss(mL)	300.00(50.00-450.00)	200.00(100.00-462.50)	-0.432	0.666
Infusion volume(L)	2.00(1.50-2.50)	2.00(1.50-3.00)	-0.239	0.811
^a^Blood transfusion(mL)	0(0-0)	0(0-400.00)	-0.549	0.853
HI-score	46.50(30.00-110.50)	56.00(30.00-105.00)	-0.568	0.570
Hemodynamic variables	6.00(0-11.75)	4.00(0-10.00)	-1.734	0.083
Volume therapy	30.00(14.00-30.00)	30.00(14.00-30.00)	-0.352	0.725
Medication	10.00(0-75.00)	35.00(-75.00)	-1.204	0.229
Postoperative outcomes				
Postoperative SBP(mmHg)	129.02 ± 22.14	123.86 ± 20.27	-1.296	0.195
Postoperative DBP(mmHg)	70.00(68.00-80.00)	75.00(65.00-85.00)	-0.669	0.503
Postoperative heart rate(bpm)	80.00(70.00-90.75)	78.50(69.50-85.25)	-0.620	0.535
Fluid intake during 24-48 hours after surgery(L)	2.24 ± 0.84	2.49 ± 1.10	2.984	0.184
Cases of admitting to ICU	2/60	3/50	0.384	0.535
Length of the ICU stay(days)	0(0-0)	0(0-0)	-0.167	0.868
Postoperative hospitalization days	7.00(5.25-9.00)	6.00(4.75-8.00)	-1.885	0.059
^b^Complications	8/60	4/50	0.084	0.772

Data are shown as mean ± SD, medians, and [P5-P95] intervals or proportions. PPGL, pheochromocytoma and paraganglioma; PXB, phenoxybenzamine; BMI, body mass index; outside the targets means at least one of SBP >160 mmHg, MAP <60 mmHg, HR >100 bpm and HR <50 bpm appears; SBP, systolic blood pressure; DBP, diastolic blood pressure; HR, heart rate; bpm, beats per minutes; HI-score, hemodynamic instability score; ICU, intensive care unit; ASA, American Society of Anesthesiologists. *Statistically significant.

a Transfusion of red blood cells and plasma.

b assessed according to the Common Terminology Criteria for Adverse Events.

## 5 Discussion

PPGL patients are usually treated preoperatively with α-blockade to reduce the likelihood of severe hemodynamic fluctuations during surgery (15). However, there is no consensus on the duration of preoperative management of α-blockade. Thus, we retrospectively analyzed the intraoperative hemodynamics and postoperative outcomes of 166 patients with PPGL at different preoperative durations of PXB and the different catecholamine secretory phenotypes, respectively. The results showed that preoperative preparation with PXB for more than 14 days might not have any additional benefits on intraoperative hemodynamic stability and postoperative outcomes. Moreover, patients with the adrenergic phenotype were more likely to have intraoperative hemodynamic instability. Plasma-free MN was an independent predictor of intraoperative hemodynamic instability in patients with PPGL.

While all guidelines recommend α-blockade as first-line therapy for hormonally secreting PPGL, the evidence is not yet strong enough to fully support this recommendation (9, 10). Previous studies (23–25) have suggested that α-blockade may improve perioperative outcomes. However, some experts have raised concerns about the potential side effects of these drugs, including hypotension and increased risk in patients with comorbid cardiovascular disease. Even so, other researchers have found no significant difference in intraoperative hemodynamic stability with or without α-blockade pretreatment (26, 27), which is consistent with our findings. Although the median total HI-score was higher in the PXB group than in the no α-blockade group, this may be due to potential biases in patient selection. As the latter group was comprised mainly of normotensive patients with mild symptoms, while the former group had more severe clinical presentations and higher pre-treatment blood pressure, requiring medical intervention more frequently. Thus, we believe that α-blockade is still necessary for patients with elevated blood pressure and overproduction of catecholamines. However, further studies are needed to determine how to individualize α-blockade pretreatment, particularly in patients with normal blood pressure and normal catecholamine levels.

In our center, most patients underwent preoperative preparation with PXB for more than seven days, as recommended by recent guidelines (9, 10). However, domestic guidelines suggest a longer preparation time of more than two weeks for safety reasons (11). Over the past few decades, there were several retrospective studies have reported the preparation duration of 16 days, 27 days, 35 days, and even 14 weeks (6, 13–15, 28). These studies covered a wide spectrum with different surgical skills, anesthesia techniques, and different definitions of intraoperative hemodynamic instability. However, the preparation time of PXB has decreased over time. A previous study of 102 patients with PPGL who were treated with PXB between 2001 and 2018 in the Department of Urology, Peking University First Hospital, found that the median preparation time with PXB was 27.4 days. The study reported no significant differences in intraoperative hemodynamics and postoperative complications among the three groups taking PXB for less than 14 days, 14-30 days, and more than 30 days (8). Similarly, in our study, we found that lengthening the duration did not result in an increase in the cumulative time outside the target blood pressure or total HI-score.

Despite 58.6% of patients in our study having intraoperative hemodynamic instability, we observed no death and a very low incidence of severe complications, which is consistent with the literature (5, 28, 29). Notably, our study found a significantly lower proportion of patients presenting with peak systolic blood pressure greater than 200mmHg compared to a study conducted at Peking University First Hospital (2.7% vs. 39.1%). Additionally, the rate of patients admitted to the ICU (6.2% vs. 76.5%) and postoperative complications (23.5% vs. 38.3%) were also significantly lower in our study than that reported by Peking University First Hospital (13). We speculated the main reason for the better outcomes in our hospital is the high proportion of laparoscopic procedures(92.4% versus 61.8%). In recent years, laparoscopic adrenalectomy has been used more frequently at our center. In fact, a previous study at our center found that between 2002 and 2017, only 77.5% of the patients underwent laparoscopic resection, compared to 92.4% in our study from December 2014 to January 2020 (30). Consequently, the incidence of intraoperative hemodynamic instability and postoperative complications was lower in our study. Similarly, laparoscopic surgery was also reported to be associated with a lower risk of intraoperative bleeding than open surgery for patients with PPGL (31). Moreover, advances in anesthesia, improved multidisciplinary management, and sufficient preparation, including a high-sodium diet and fluid intake, as well as effective medical treatments for comorbidities, likely also contributed to improved perioperative outcomes. With these advancements, we speculate that the preparation time could be further reduced while still ensuring perioperative hemodynamic stability.

In our center, the median duration of PXB preparation for patients was 18 days. Only one-third were prepared for less than 2 weeks, with a median duration of 13 days. Some patients took PXB for longer than necessary because of late attendance at the clinic, but all achieved the necessary targets and remained stabilized before surgery. Our study found that patients who were managed with PXB for over 21 days had a reduced risk of intraoperative hypotension, as well as shorter hospital and ICU stays. Long-term treatment with PXB leads to receptor hypersensitization, which enhances the vasoconstriction caused by α-adrenoceptors (32). However, any intraoperative hypotension that lasts only a short time can be easily corrected with volume repletion and vasoactive drugs. Further exploration of this benefit is warranted if the preoperative administration with PXB needs to be extended beyond 21 days. Our findings suggested that achieving sufficient preparation targets may be more important than preparing for a fixed duration. And the preparation time should be personalized to the individual patient.

Hemodynamic instability in patients with PPGL is primarily caused by the diversity of levels and types of catecholamines secreted. There are two kinds of secretory phenotypes, adrenergic and noradrenergic. Patients of the noradrenergic phenotype in our study were younger, had smaller tumor diameters, and were more likely to have extra-adrenal tumors, as reported in most studies (33–37). Moreover, patients with the adrenergic phenotype had a higher probability in frequency and cumulative time of intraoperative hemodynamic instability than the noradrenergic phenotype. Both norepinephrine and epinephrine can act on α-adrenoceptors and result in hypertension due to peripheral vasoconstriction and increased peripheral resistance. As opposed to norepinephrine, epinephrine stimulates the function of the β1-adrenoceptors, enhancing the contraction and elevating the heart rate. In 90% of tumors with the adrenergic phenotype, plasma-free MN and NMN were elevated, which may explain the stronger blood pressure-raising effect compared to tumors with the noradrenergic phenotype. Conversely, recent research has shown that patients with the adrenergic phenotype tend to present with paroxysmal hypertension, while patients with the noradrenergic phenotype usually have more severe and sustained hypertension. Thus, patients with the noradrenergic phenotype are at greater risk of hemodynamic instability (6, 33). There was a larger tumor size and a higher level of plasma-free MN in patients with intraoperative hemodynamic instability than in patients without. Multivariate analysis showed that only plasma-free MN was associated with intraoperative hemodynamic instability, and all PPGL with elevated plasma-free MN belonged to the adrenergic phenotype. In other studies, tumor size and surgery methods were also found to be associated with intraoperative hemodynamic instability (11, 13, 38). As the size of the tumor increases, so does the hormone secretion, which may indirectly affect hemodynamics *via* catecholamine release. Hence, more attention should be given to preventing intraoperative blood pressure fluctuations in PPGLs with predominant adrenaline secretion.

Finally, there are no standardized criteria to evaluate hemodynamic instability, which can make comparisons between studies difficult. Some studies used the maximum and minimum blood pressures, which only represent a single point in time. In our study, intraoperative hemodynamic instability was defined as SBP >160 mmHg, MAP <60 mmHg, HR >100 bpm, and HR <50 bpm, as these are clinically recognized thresholds for intervention (29, 39). Furthermore, we believe that the cumulative time spent outside these targets may be a better indicator of hemodynamic fluctuations. To further illustrate the severity of intraoperative hemodynamic instability, we also included the maximum and minimum blood pressure and heart rate readings. Consequently, our study was more comprehensive and quantitative in terms of assessing the perioperative outcomes. Moreover, we utilized the HI-score, a validated and novel scoring system for grading intraoperative hemodynamic instability that incorporates hemodynamic variables and treatment measures. This scoring system includes routine measurements of surgical procedures with a certain risk of hemodynamic instability during general anesthesia (29). With such a comprehensive index to evaluate hemodynamic instability, our study was able to make a relatively convincing conclusion. However, we still anticipate the need for prospective randomized multicenter studies to make a final conclusion in the future.

Our study has several advantages, including a relatively large sample size for a tumor with low prevalence, comprehensive data collection, and detailed subgroup analysis. With more than 20 years of experience in laparoscopy with highly dedicated and experienced operators and anesthesiologists, the impact of the duration of α-blockade pre-treatment on intraoperative hemodynamics was less evident than before. Also, there are several limitations to our study. First, we did not perform further subgroup analysis for patients who prepared less than 14 days in advance. Therefore, we were unable to explore if the shorter time of less than 14 days, for example, 7 days, would be sufficient if the patient had reached the necessary preparation targets. Second, serum dopamine and 3-MTY are newly launched routine projects in our center and only a few cases were measured for these items. These dopamine-secreted PPGL which only secret dopamine but not adrenaline and noradrenalin might have a different pattern of intraoperative hemodynamic variation which is worthy of further study. Therefore, we were unable to demonstrate how dopamine influences hemodynamic instability. Third, as a retrospective monocentric study, it is inevitable to have selective and recall bias, which makes it difficult to extend the findings to clinical practice. Finally, few patients had genetic testing, so we could not access the impacts of genotypes on secretory phenotypes and intraoperative hemodynamics.

In conclusion, this study investigated the impact of the duration of PXB treatment and secretory phenotypes on intraoperative hemodynamics and postoperative outcomes in patients with PPGL. As PXB has been shown to have the comparable effects of maintaining intraoperative hemodynamic stability with other α-blockade (39), we can conclude that preparing with α-blockade for nearly fourteen days is sufficient to ensure intraoperative hemodynamic stability in PPGL, especially, under laparoscopic resection with a dedicated team of anesthesiologists. The level of plasma-free MN is the most important independent risk factor for intraoperative hemodynamic instability, highlighting the need for increased attention to patients with predominant adrenaline secretions. Additionally, our study found a very low incidence of postoperative complications with the application of laparoscopy and advanced anesthesia, suggesting that the preparation time could potentially be shortened if sufficient preparation targets are met.

## Data availability statement

The original contributions presented in the study are included in the article/**Supplementary Material**. Further inquiries can be directed to the corresponding authors.

## Ethics statement

The studies involving human participants were reviewed and approved by Institutional Review Board at Sun Yat-sen Memorial Hospital, Sun Yat-sen University. The patients/participants provided their written informed consent to participate in this study.

## Author contributions

YY, SZ, and TL designed the study and critically revised the manuscript. YY and JF collected data. YY and YG performed data analysis and drafted the manuscript. SZ and LW reviewed and edited the manuscript. All authors contributed to the article and approved the submitted version.
