# Supplementary material for: Influence of duration of preoperative treatment with phenoxybenzamine and secretory phenotypes on perioperative hemodynamics and postoperative outcomes in pheochromocytoma and paraganglioma
Source: Front Endocrinol (Lausanne). 2023 Apr 19;14:1139015. doi: 10.3389/fendo.2023.1139015 (PMC10154584; doi:10.3389/fendo.2023.1139015)
Supplement: Supplementary file 1 [file Table_1.docx]

Supplementary Material

Influence of duration of preoperative treatment with phenoxybenzamine(PXB) and secretory phenotypes on perioperative hemodynamics and postoperative outcomes in pheochromocytoma and paraganglioma(PPGL)

^[[1]](#footnote-1)^Yao Yao^1^, Ying Guo^1^, Jing Fan^1^, Tianxin Lin^2^, Lin Wang^3*^, Shaoling Zhang^1*^

^1^Department of Endocrinology, Sun Yat-sen Memorial Hospital, Sun Yat-sen University, Guangzhou, China

^2^ Department of Urology, Sun Yat-sen Memorial Hospital, Sun Yat-sen University, Guangzhou, China

^3^ Department of Pathology, Sun Yat-sen Memorial Hospital, Sun Yat-sen University, Guangzhou, China

* Correspondence:
zhshaol@mail.sysu.edu.cn; wanglin5@mail.sysu.edu.cn

Supplement table 1. Threshold values for all hemodynamic instability score components

| Domains | HI-score components | Value | Score |
| --- | --- | --- | --- |
| Hemodynamic variables | Maximum SAP (mmHg) | <160 | 0 |
|  |  | 160 to 179 | 1 |
|  |  | 180 to 199 | 3 |
|  |  | ≥200 | 7 |
|  | Time SAP > 160 mmHg (%) | 0 | 0 |
|  |  | 0.1 to 1.0 | 1 |
|  |  | 1.1 to 6.6 | 3 |
|  |  | ≥6.7 | 7 |
|  | Minimum MAP (mmHg) | ≥60 | 0 |
|  |  | 50 to 59 | 1 |
|  |  | 40 to 49 | 3 |
|  |  | <40 | 7 |
|  | Time MAP < 60 mmHg (%) | 0 | 0 |
|  |  | 0.1 to 1.1 | 1 |
|  |  | 1.2 to 4.1 | 3 |
|  |  | ≥4.2 | 7 |
|  | Maximum HR (bpm) | <100 | 0 |
|  |  | 100 to 119 | 1 |
|  |  | ≥120 | 3 |
|  | Time HR > 100 bpm (%) | 0 | 0 |
|  |  | 0.1 to 1.0 | 1 |
|  |  | >1.0 | 3 |
|  | Minimum HR (bpm) | ≥50 | 0 |
|  |  | 40-49 | 1 |
|  |  | <40 | 3 |
|  | Time HR < 50 bpm (%) | 0 | 0 |
|  |  | 0.1-1.7 | 1 |
|  |  | >1.7 | 3 |
| Volume therapy | Volume therapy(ml/kg/h) | 0 to 84ml/h | 0 |
|  |  | <6.3 | 2 |
|  |  | 6.4-9.7 | 6 |
|  |  | 9.8-14.3 | 14 |
|  |  | >14.3 | 30 |
| Cardiovascular medication | Norepinephrine(μg/kg/h) | 0 |  |
|  |  | >0 to 1.48 | 5 |
|  |  | 1.49 to 2.47 | 15 |
|  |  | 2.48 to 4.14 | 35 |
|  |  | >4.14 | 75 |
|  | Phenylephrine (μg/kg/h) | 0 | 0 |
|  |  | >0.0 to 2.06 | 4 |
|  |  | >2.06 | 12 |
|  | Dobutamine (mg/kg/h) | 0 | 0 |
|  |  | >0 to 0.22 | 1 |
|  |  | >0.22 | 3 |
|  | Total |  | 160 |

HI-score, hemodynamic instability score; HR, heart rate; MAP, mean arterial pressure; SAP, systolic arterial pressure.

Supplement table 2. American Society of Anesthesiologists Classification

| ASA class | Definition |
| --- | --- |
| Ι | Healthy patient |
| Ⅱ | Mild to moderate system diseases caused by the surgical condition or by other pathological processes, and medically well controlled |
| Ⅲ | Severe disease process which limits activity but is not incapacitating |
| Ⅳ | Severe incapacitating disease process that is a constant threat to life |
| Ⅴ | Moribund patient not expected to survive 24 hours with or without the operation |
| Ⅵ | Declared brain-dead patients whose organs are being removed for donor purpose |

ASA, American Society of Anesthesiologists.

Supplement table 3. Characteristics of patients with PPGL between the PXB group and no α-blockade group

|  | no α-blockade(n=21) | PXB(n=145) | *t/Z*/*χ*^2^ | *p* value |
| --- | --- | --- | --- | --- |
| Sex ratio(male/female) | 11/10 | 78/67 | 0.015 | 0.903 |
| Median age at diagnosis(years) | 48.76±15.51 | 48.15±15.44 | 0.169 | 0.866 |
| Median tumor diameter (cm) | 5.50(4.25-7.00) | 4.70(3.80-6.50) | -1.095 | 0.274 |
| Preoperative SBP(mmHg) | 120.00(109.50-124.00) | 133.00(120.00-149.50) | -3.243 | 0.001^*^ |
| Preoperative DBP(mmHg) | 78.00(72.50-83.50) | 83.00(73.00-93.50) | -1.903 | 0.057 |
| Preoperative HR(bpm) | 80.00(70.00-95.25) | 81.00(75.00-93.00) | -0.626 | 0.531 |
| ASA class Ⅱ/Ⅲ/Ⅳ(n) | 10/11/0 | 22/113/10 | 12.021 | 0.002^*^ |
| ^a^Anesthesia duration(minutes) | 280.00(231.50-385.00) | 280.00(215.00-331.50) | -0.566 | 0.571 |
| ^b^Surgery duration(minutes) | 130.00(92.50-192.50) | 125.00(80.00-190.00) | -0.403 | 0.687 |

Data is shown as mean ± SD, medians and [*P*5-*P*95] intervals or proportions. PPGL, pheochromocytoma and paraganglioma; PXB, phenoxybenzamine; BMI, body mass index; SBP, systolic blood pressure; DBP, diastolic blood pressure; HR, heart rate; bpm, beats per minutes; ASA, American Society of Anesthesiologists. ^*^Statistically significant.

^a^Time from incision until suturing of the incision.

^b^Time from induction of anesthesia until suturing of the incision.

Supplement table 4.Characteristics of patients with PPGL according to the occurrence of intraoperative hemodynamic instability or not.

|  | Intraoperative hemodynamic instability(n=96) | No Intraoperative hemodynamic instability(n=70) | *t*/*z*/*χ*^2^ | P value |
| --- | --- | --- | --- | --- |
| Sex ratio(male/female) | 37/59 | 40/30 | 5.632 | 0.018^*^ |
| BMI(kg/m2) | 21.72±3.24 | 22.91±3.29 | 0.329 | 0.568 |
| Mean age at diagnosis(years) | 48.6±16.1 | 47.7±14.6 | 0.024 | 0.876 |
| Diabetes(n) | 31/65 | 17/53 | 1.262 | 0.261 |
| Paroxysmal symptoms | 55/41 | 27/43 | 5.676 | 0.017^*^ |
| Tumor location(adrenal/extra-adrenal) | 69/27 | 58/12 | 2.716 | 0.099 |
| Mean tumor diameter (cm) | 5.00(3.80-7.00) | 4.50(3.75-6.00) | -2.370 | 0.018^*^ |
| Plasma MN(nmol/L) | 1.91(0.27-8.87) | 0.34(0.16-2.13) | -3.543 | <0.001^*^ |
| Plasma NMN(nmol/L) | 8.27(2.10-18.57) | 6.86(1.59-11.25) | -1.892 | 0.058 |
| Median time of administration of PXB(days) | 18.00(14.00-26.50) | 18.50(14.00-28.50) | -0.224 | 0.822 |
| Total dosage of PXB(g) | 0.62(0.44-0.98) | 0.63(0.39-0.98) | -0.586 | 0.558 |
| Surgery approach  (laparoscopy/open surgery) | 85/11 | 69/1 | 6.072 | 0.014^*^ |
| Transabdominal/retroperitoneal | 82/14 | 59/11 | 0.004 | 0.841 |
| ^a^Surgery time(minutes) | 122.50(90.00-200.00) | 125.00(80.00-190.00) | -1.442 | 0.149 |
| ASA class Ⅱ/Ⅲ/Ⅳ(n) | 14/77/5 | 18/47/5 | 3.778 | 0.151 |
| ^b^Anesthesia duration(minutes) | 300.00(227.25-377.50) | 257.50(210.00-310.00) | -3.177 | 0.001^*^ |
| Preoperative SBP(mmHg) | 131.50(120.00-150.00) | 130.00(118.50-141.00) | -0.795 | 0.427 |
| Preoperative DBP(mmHg) | 80.00(72.00-93.75) | 84.00(73.75-90.25) | -0.352 | 0.725 |
| Preoperative HR(bpm) | 80.00(72.00-93.00) | 81.00(78.00-93.25) | -0.461 | 0.645 |
| antihypertensive drugs |  |  |  |  |
| Frequency(n) | 71 | 33 | 12.440 | <0.001^*^ |
| Number(n) | 1.00(0-2.00) | 0(0-1.00) | -4.145 | <0.001^*^ |
| Vasoactive drugs |  |  |  |  |
| Frequency(n) | 61 | 26 | 11.311 | 0.001^*^ |
| Number(n) | 1.00(0-1.00) | 0(0-1.00) | -3.377 | 0.001^*^ |
| Length of the ICU stay(days) | 0(0-0) | 0(0-0) | -2.130 | 0.033 |
| Postoperative hospitalization days | 7.00(5.25-9.00) | 6.00(5.00-7.00) | -2.735 | 0.006^*^ |

Data are shown as mean ± SD, medians and [*P*5-*P*95] intervals or proportions. PPGL, pheochromocytoma and paraganglioma; PXB, phenoxybenzamine; BMI, body mass index; SBP, systolic blood pressure; DBP, diastolic blood pressure; HR, heart rate; bpm, beats per minutes; HI-score, hemodynamic instability score; ICU: intensive care unit; ASA, American Society of Anesthesiologists. *Statistically significant.

^a^Time from incision until suturing of the incision.

^b^Time from induction of anesthesia until suturing of the incision.

Supplement table 5. Characteristics of patients with PPGL according to the occurrence of postoperative complications instability or not.

|  | Postoperative complications(n=24) | No postoperative complications(n=142) | *t*/*z*/*χ*^2^ | P value |
| --- | --- | --- | --- | --- |
| Sex ratio(male/female) | 12/12 | 65/77 | 0.147 | 0.701 |
| BMI(kg/m2) | 22.31±3.66 | 22.23±3.48 | -0.091 | 0.928 |
| Mean age at diagnosis(years) | 52.08±15.74 | 47.49±15.28 | -1.355 | 0.177 |
| Diabetes(n) | 11/13 | 37/105 | 3.907 | 0.048^*^ |
| Paroxysmal symptoms | 12/12 | 70/72 | 0.004 | 0.949 |
| Tumor location(adrenal/extra-adrenal) | 18/6 | 109/22 | 0.035 | 0.851 |
| Mean tumor diameter(cm) | 5.90(4.60-7.00) | 4.55(3.75-6.50) | -2.178 | 0.229 |
| Plasma free MN(nmol/L) | 1.89(0.33-5.75) | 0.76(0.19-3.94) | -1.358 | 0.174 |
| Plasma free NMN(nmol/L) | 5.29(1.77-17.03) | 7.45(2.22-14.74) | -0.427 | 0.669 |
| Median time of administration of PXB(days) | 19.00(15.00-30.50) | 17.50(13.25-26.75) | -1.229 | 0.219 |
| Total dosage of PXB(g) | 900.00(490.00-1515.00) | 600.00(407.50-900.00) | -1.258 | 0.208 |
| Surgery approach  (laparoscopy/open surgery) | 21/3 | 133/9 | 1.004 | 0.316 |
| Transabdominal/retroperitoneal | 19/5 | 122/20 | 0.678 | 0.410 |
| ^a^Surgery time(minutes) | 170.00(95.00-242.50) | 122.50(80.00-180.00) | -0.220 | 0.826 |
| ASA class Ⅱ/Ⅲ/Ⅳ(n) | 6/17/1 | 26/107/9 | 0.681 | 0.409 |
| ^b^Anesthesia duration(minutes) | 312.50(227.50-456.00) | 273.50(215.00-330.00) | -1.732 | 0.083 |
| Preoperative SBP(mmHg) | 124.50(110.00-143.00) | 131.0(120.00-149.25) | -1.351 | 0.177 |
| Preoperative DBP(mmHg) | 82.00(71.25-92.25) | 82.00(73.00-93.00) | -0.280 | 0.779 |
| Preoperative HR(bpm) | 80.50(75.75-101.75) | 81.00(74.75-93.00) | -0.576 | 0.565 |
| Blood loss(mL) | 95.00(50.00-575.00) | 150.00(25.00-325.00) | -0.398 | 0.691 |

Data is shown as mean ± SD, medians and [*P*5-*P*95] intervals or proportions. PPGL, pheochromocytoma and paraganglioma; BMI, body mass index; SBP, systolic blood pressure; DBP, diastolic blood pressure; HR, heart rate; PXB, phenoxybenzamine; ICU: intensive care unit; ^*^Statistically significant.

^a^Time from incision until suturing of the incision.

^b^Time from induction of anesthesia until suturing of the incision.

1. *Correspondence: [zhshaol@mail.sysu.edu.cn;](mailto:zhshaol@mail.sysu.edu.cn;) wanglin5@mail.sysu.edu.cn

   Department of Endocrinology, Sun Yat-sen Memorial Hospital, Sun Yat-sen Memorial University, 107 Yanjiang West Road, Guangzhou 510120, China [↑](#footnote-ref-1)
